# Indications for an antidepressive effect of thymosin alpha-1 in a small open-label proof of concept study in common variable immune deficiency patients with depression

**DOI:** 10.1016/j.bbih.2024.100934

**Published:** 2025-01-02

**Authors:** Daniël G. Aynekulu Mersha, Sarah E. Fromme, Frank van Boven, Gara Arteaga-Henríquez, Annemarie Wijkhuijs, Marianne van der Ent, Raf Bergmans, Bernard T. Baune, Hemmo A. Drexhage, Virgil Dalm

**Affiliations:** a Dept of Immunology, Erasmus Medical Center, Rotterdam, the Netherlands; b Dept of Psychiatry, University of Muenster, Muenster, Germany; c Department of Internal Medicine, Section of Allergology & Clinical Immunology, Erasmus MC, University Medical Center Rotterdam, P.O. Box 2040, 3000, CA, Rotterdam, the Netherlands; d Department of Mental Health, Hospital Universitari Vall d'Hebron, Barcelona, Catalonia, Spain; e Group of Psychiatry, Mental Health, and Addictions, Vall d'Hebron Research Institute (VHIR), Barcelona, Catalonia, Spain; f Biomedical Network Research Center on Mental Health (CIBERSAM), Barcelona, Catalonia, Spain; g The Florey Institute of Neuroscience and Mental Health, Melbourne, Australia; h Department of Psychiatry, University of Melbourne, Melbourne, Australia

**Keywords:** CVID, Depression, Thymalfasin, Therapy, Improvement

## Abstract

**Background:**

A considerable proportion (21%) of patients with common variable immunodeficiency (CVID) suffers from depression. These subjects are characterized by reduced naïve T cells and a premature T cell senescence similar to that of patients with major depressive disorder (MDD). It is known that T cells are essential for limbic system development/function. Treatment with thymosin α1 (Tα1) is capable to increase the thymus output of naïve T cells.

**Objective:**

To treat CVID patients with a comorbid depressive episode with Tα1 to increase naïve T cells and thereby improve mood.

**Design:**

A small open-label, proof of concept trial. Five depressed CVID patients (Hamilton Depression Rating Scale, HDRS >12) could be treated with Tα1 (8 weeks, 1.6 mg daily subcutaneously, 1st week, thereafter 1.6 mg twice weekly). At the start, at 8 weeks and 8 weeks after the last injection, the HDRS was recorded and blood samples drawn for measuring naïve and memory T cells, Th17 and Treg cells, hsCRP, IL-6 and IL-7. Outcomes were compared to those of a contrast group (42 MDD patients, same severity but treated as usual (TAU)).

**Results:**

In all 5 depressed CVID patients HDRS decreased during Tα1 treatment (with average 52%, TAU decreased scores with 36% in MDD patients). All 5 CVID patients showed an increase in naïve/memory CD4^+^ and CD8^+^ T cell ratios, and in 4 of the 5 patients with detectable IL-6 levels reductions were recorded. TAU did not show such immune improvements. In the 8-week wash-out, depression recurred in the 2 most severe patients, while continued to improve in the others. Immune effects were not sustained in the wash-out.

**Conclusion:**

This preliminary small study suggests thymus hormone treatment to have antidepressive and related immune correcting effects. Data urge for larger placebo-controlled trials.

## 1 Introduction

Common variable immunodeficiency (CVID) is one of the most prevalent primary immunodeficiency diseases. It is characterized by recurrent infections and a deficient production of immunoglobulins due to severe B cell deficits (Primary immunodeficiency diseases, 2007). Apart from these severe B cell deficits, T cell function is also often affected (Manusama et al., 2023). This defective T cell function is seen as one of the reasons for the comorbidities in CVID patients, such as various autoimmune diseases and allergies.

We recently confirmed the abnormal T cell function in CVID by showing in a group of 52 patients of our outpatient department, a decreased frequency of CD4^+^ T helper cells, mainly due to decreases in naïve CD4^+^ T helper cells. In contrast, frequencies of CD8^+^ T memory populations were increased, mainly due to increases in CD8^+^ TEM, TEMRA and senescent (CD57^+^) TEMRA cells (Manusama et al., 2023). Findings were interpreted as an illustration of a premature senescence of the T cell system in CVID.

Since premature T cell senescence with similar impaired generation of CD4^+^ naïve T cells and overproduction of CD4^+^ and CD8^+^ memory T cells is also a characteristic of patients with Major Depressive Disorder (MDD) (Simon et al., 2023), we investigated whether depression was more prevalent in our group of outpatients with CVID and correlated to T cell senescence characteristics. Using the Four-Dimensional Symptom Questionnaire (4DSQ), we found that a considerable proportion (21%) of the patients with CVID suffered from depression, compared to 6% of the general population using the same quantification instruments (Manusama et al., 2022). We also established that the depressed CVID patients were indeed characterized by a T cell immunotype that was similar to that of MDD patients with strong decreases in naïve CD4^+^ T helper cells.

How does one envisage such deficiencies of naïve CD4^+^ T cells to be responsible for abnormal function and morphology of the “emotional” brain? Apart from being important players in the regulation of cells of the immune system, T cells (and in particular CD4+T helper cells) are essential for normal limbic system development and function. T cell deficient rodents show reduced hippocampal neurogenesis, anxious behavior and impaired learning and a decreased brain-derived neurotrophic factor (BDNF) expression in the brain (Clark et al., 2016). Transfer of CD4^+^ T cells to these animals restores these behavioral abnormalities (Clark et al., 2016; Laumet et al., 2020; Niebling et al., 2014). In particular CD4^+^ Th17 and Treg cells play an important role in balancing mood in these preclinical models (Kim et al., 2021). A special entry route for these CCR6+CD4^+^ T helper cells has been detected via the choroid plexus, where CCL20+ microglia attracts these T cells (Sallusto et al., 2012). While infiltration of Th17 cells generally induces fear and anxiety, T regulatory cells dampen such emotions (Park et al., 2022). Chronic forms of unavoidable stress induce increased Th17 activity, in some models both in the gut and the brain, illustrating the involvement of the gut-brain axis in chronic stress induced mood regulation (Westfall et al., 2021; Medina-Rodriguez et al., 2023). Mechanisms via which such brain infiltrating CD4^+^ T helper subsets influence mood are not yet clear. The immune cells might regulate (non-mutually exclusive) the production of BDNF and neurogenesis, and/or the inflammatory state of microglia in the limbic system (Clark et al., 2016). Pro-inflammatory activation of limbic system microglia is a known phenomenon in major depression (Culmsee et al., 2019), and this so-called state of low grade brain inflammation is systemically reflected by raised blood levels of proinflammatory cytokines (amongst which IL-6, 14).

Here we investigate whether treatment with thymus hormone corrected the premature T cell senescence of depressed patients with CVID, changed the Th17/Treg balance and serum cytokine levels and improved mood. At an earlier occasion our group reported a study (Tas et al., 1990) in which a thymus hormone preparation (TP-1, SERONO) was given to patients with chronic and recurrent upper respiratory tract infections and T cell defects (reduced skin tests to recall antigens, reduced macrophage migration inhibitory factor production), reminiscent to the T cell defects described here. These patients were also characterized by a low mood and energy loss. TP-1-treatment not only resulted in improvement of the immune and infectious problems, but also clearly improved mood and energy. These former results were the impetus of the here reported study to evaluate the capability of thymus hormone to correct the reduction in naïve CD4^+^ T cells and low mood in the depressed CVID patients.

TP-1 is of the market, but Thymosin α1 (Thymalfasin) is a thymus hormone preparation currently available from Sciclone. It has multiple mechanisms of action, including direct stimulation of precursor T cells, stimulation of antigen presenting cells via Toll-Like Receptors (TLRs), reduction of apoptosis and stimulation of thymopoetic cytokines such as IL-7 and IL-15 (King and Tuthill, 2016).

The aim of this study was therefore to investigate if treatment with thymosin α1 was effective in correcting the premature T cell senescence in patients with CVID under a comorbid major depressive episode (primary objective). We also investigated the effectiveness of thymosin α1 in improving mood (secondary objectives).

Relevant for the here described studies is that Liu et al. (2020) and Jia et al. (2015) showed a thymosin α1 increased output of naïve CD4^+^ T cells from the thymus in patients with COVID and COPD respectively. Also, in HIV infection thymosin α1 treatment has been shown to increase the number of CD4^+^ T helper cells when given in combination with interferon-α and an antiviral agent (Matteucci et al., 2017).

We conducted an open-label, proof of concept trial (EudraCT number 2017-005001-12).

We were able to include five depressed CVID patients. These patients were treated with thymosin α1 for a total of eight weeks (this was the time frame effective in our previous study with TP-1, 15). Prior to the start of treatment, eight weeks after initiation of treatment, and eight weeks after the last injection of study medication (wash out period) blood samples were drawn particularly for evaluation of naïve and (senescent) T memory lymphocyte subset patterns. Moreover, various questionnaires were taken at the three time-points, notably the Hamilton Depression Rating Scale (HDRS) and the Fatigue Severity Scale (FSS). Further additional outcomes included changes in the Th17/Treg balance, the level of the inflammatory markers hs-CRP and IL-6, the T cell growth factor IL-7 and of sCD25, a marker of T cell activity.

The medical ethical committee of ErasmusMC allowed us to conduct only an open-label, proof of concept trial regarding Thymosin α1. We were fortunate to conduct at the same time a study in which a group of major depressed patients was included who were tested using the same immune assays; this group which was treated as usual (TAU) and served as a control group for patients which were conducting spinning therapy as add-on to TAU. We used the MDD-TAU group as contrast group for this study and the inclusion of this contrast group enabled us to compare outcomes of the changes in T cell profiles, serum analytes and depression scores of the CVID patients to those of the contrast group of MDD patients with the same severity of depression, but treated as usual (TAU). The contrast group was sampled within the same time frame at the Department of Psychiatry, University of Muenster. Samples of both studies were simultaneously tested at the laboratory of the Department of Immunology, ErasmusMC University Medical Center, Rotterdam. As a result, we combined both groups (CVID and MDD-TAU) into a post hoc non-randomized open trial aiming to investigate whether the treatment of mood co-morbidity in CVID with thymosin α1 is comparable to treatment as usual in patients with MDD, and whether there were signs of correction of premature T cell senescence and other immune parameters in the treatment groups.

## 2 Patients and methods

### 2.1 Patients

*Patients with CVID and a comorbid major depressive episode (CVID-MDD patients)*. Adult outpatients with a proven diagnosis of CVID according to the International Union of Immunological Societies (IUIS) and with a depressive episode were recruited at the Primary Immunodeficiency Center of the Department of Internal Medicine, Section of Allergy and Clinical Immunology, Erasmus MC University Medical Center, Rotterdam, the Netherlands between July 2022 and February 2023. The presence of depression in patients with CVID was determined from medical records and patients with a score on the Hamilton Depression scale (17-item-version, HDRS-17) of at least 12 were eligible for inclusion. Patients were at the time of testing free of uncontrolled disease.

Five patients could be included in the study in the indicated study time frame. Table 1 gives socio-demographic data, clinical data and base-line immune abnormalities of the 5 patients.

**Table 1:** Baseline clinical and immune characteristics of the included CVID patients with a depression.

	Patient 1	Patient 2	Patient 3	Patient 4	Patient 5	CVID (all)	MDD TAU	HC
**Number**						5	42	20
**Age (yrs)**	51	44	42	38	59	47 (Niebling et al., 2014)	35 (Medina-Rodriguez et al., 2023)	31 (Medina-Rodriguez et al., 2023)
**Sex**	M	M	F	F	M	3M, 2F	22M,20F	8M, 12F
**HAM-D at baseline**	24	25	14	16	14	19 (Clark et al., 2016)	20 (Manusama et al., 2022)	2 (Manusama et al., 2023)
**FFS at baseline**	60	49	49	58	41	51 (Niebling et al., 2014)	ND	ND
**Anti-depressants**	Olanzapine, Sertraline, Benzo	Vortioxetine	No	No	No	2/5	40% not medicated, 30% one antidepressant, 30% more than one	No
**Psychotherapy**	Yes	No (past)	No	No	No	1/5	All	No
**Routine lymphocyte subsets**	CD8+↑ B↓	CD8+↑ B↓	NK ↓	B↓ NK↓	B↓	4/5 B cells decreased	Not tested Literature: CD8 increased, NK decreased	
**Senescent CD8^+^Cells**	Naive/Memory ↓	Naive ↓ Memory ↑ (EMRA) Naive/Memory ↓ CD57^+^ Memory ↑				2/5	28% of the cohort	
**Senescent CD4^+^Cells**	Naive ↓ Naive/Memory ↓	Naive ↓ Naive/Memory ↓ CD57^+^ Memory ↑				2/5	28% of the cohort	
**Functional T helper Subsets**	Th2 ↑	Th1↑ Th2↑ T reg↓	Th2↑ T reg↑	Th2↑	Th1↑ Th2↑	5/5 Th2 cells increased	Not abnormal	
**Cytokines (IL-6, hsCRP, IL-7, sCD25)**	IL-6 ↑	IL6 ↑				2/5 increased IL-6	Not abnormal Literature: IL-6 increased in most studies	

With regard to the socio-demographic data, the age of the five patients ranged between 38 and 59 years and three were male. All received Immunoglobulin (Ig) therapy, all had recurrent infections, but only patient 2 and 3 had an active infection at inclusion and were on antibiotics during the thymosin treatment. None of the 5 patients suffered from an autoimmune disease or a hematological malignancy. None of the patients used anti-inflammatory drugs, including glucocorticoids.

*Patients with MDD treated as usual (*10.13039/501100004375TAU*)* were recruited in the MOODSTRATA study, which is part of the EU-funded MOODSTRATIFICATION study, at the University Hospital Münster in Germany between February 2021 and June 2023. All inpatients were diagnosed according to the Diagnostic and Statistical Manual of Mental Disorders (DSM)-5, using the Structured Clinical Interview for DSM-5 Axis I Disorders and the MINI Version 5.0.0. Included were patients with an interview confirmed MDD, and with a minimum score of 12 on the HDRS-17. Patients were (partially) hospitalized in an open station at the time of the measurements.

The average ages of the MDD group receiving TAU and of the HC were 35 (standard deviation, 12) and 31 (standard deviation, 12) years respectively, and 52% and 40% were male respectively (See Table 1). They received treatment as usual (TAU) including psychotherapy, pharmacotherapy and therapies consisting of arts and exercise.

*Healthy sex- and aged-matched individuals* (recruits through notices and advertising) collected at the University Hospital Münster, Germany in the same time period were used as controls. All healthy controls were in self-declared health, without any signs of depression or any other psychiatric condition.

*Ethics*. The study protocol was approved by the Medical Ethics Committee of the Erasmus MC University Medical Center, Rotterdam (MEC-2013-026) and of the University Hospital Münster (Münster, Germany) (2020-649-f-S IMMUNOSTRATA). All subjects provided written informed consent and the study was carried out in accordance with the Declaration of Helsinki.

### 2.2 Intervention

**Fig. 1:**
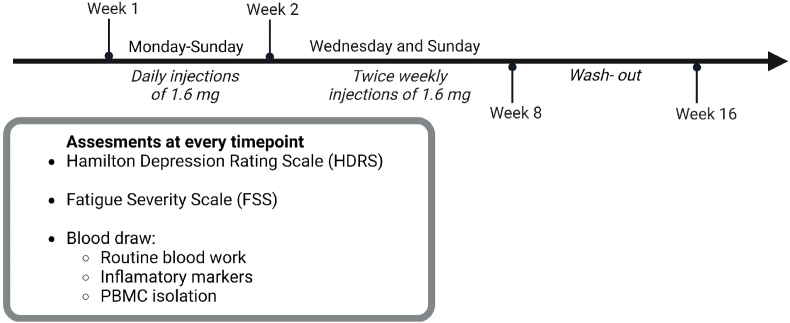
The treatment schedule of the 5 depressed CVID patients. (Created in BioRender. 4, V (2024) https://BioRender.com/a16k591).

An open-label, proof of concept trial (EudraCT number 2017-005001-12). Patients received Thymosin-α1 (Tα1, thymalfasin, Zadaxin, SciClone Pharmaceuticals, Sjanghai, China) 1.6 mg subcutaneously every day (first week) and thereafter twice a week for a consecutive seven weeks (in total eight weeks). The study design for thymalfasin treatment is given in Fig. 1.

### 2.3 Blood collection and preparation

Blood was collected in clotting tubes for serum preparation (stored at −80 °C) and in sodium–heparin tubes for immune cell preparation. Peripheral blood mononuclear cells (PBMC) were isolated by low-density gradient centrifugation shortly after blood draw, as described in detail before (Knijff et al., 2006). Isolated PBMC were frozen in 10% dimethylsulfoxide and stored in liquid nitrogen. This procedure enabled us to test patient and control samples in the same series of experiments.

### 2.4 Lymphocyte immunophenotyping

*Staining A (Routine immune determinations)*, the absolute counts of total leukocytes (CD45^+^), Natural Killer (NK) cells (CD3^−^CD16^+^CD56^+^), B-lymphocytes (CD19^+^), T-lymphocytes (CD3^+^), CD4^+^ T-lymphocytes, and CD8^+^ T-lymphocytes were determined with a clinical laboratory ISO 15189 accredited method. Staining was performed on whole blood using multitest 6-color T-B-NK reagent (BD Biosciences). Flowcytometric analyses were performed using a FACS Lyric instrument (BD Biosciences).

*Staining B (Functional T helper cell subset determinations).* Percentages of functional T helper cell subsets (Th1, Th2, Th17, Tregs) were determined using an 8-color (membrane and intracellular) staining. PBMC were thawed and the recovery and viability of cells were 73% and 95% respectively, as assessed by Trypan blue staining. A total of 1 × 10^6^ of defrosted PBMC were stimulated for 4 h at 37 °C in RPMI-1640 culture medium with 50 ng/ml phorbol 12-myristrate 13-acetate (PMA; Sigma Aldrich, St. Louis, MO, USA) and 1.0 μg/ml ionomycin (Sigma) in the presence of Golgistop (BD Biosciences). 200.000 events were collected on a BD FACS Canto II-3 laser instrument. Analysis was performed using FlowJo software (Gating strategy is given in 21). T helper cell subsets were identified by their secreting cytokines: Th1 (CD3^+^CD4+IFNγ+), Th2 (CD3^+^CD4+IL4+), Th17 (CD3^+^CD4+IL17A+). Tregs were identified by their transcription factor FOXP3 (CD3^+^CD4^+^CD25hiFOXP3+). We also measured proportions of memory CD4^+^ T cells (CD3^+^CD4^+^CD45RO+) and naïve CD4^+^ T cells (CD3^+^CD4^+^CD45RO-); the latter was indicated by subtracting the proportion of memory CD4 T cells from the total T helper lymphocytes. T cell subsets of staining B were expressed as percentages of total lymphocytes, which could be reliably detected as a clear population in scatter properties after the 4-h culture.

*Staining C (Differentiation of T helper and T cytotoxic subsets)* – For more profound analysis of T helper and cytotoxic naïve/memory cell subsets a second vial of PBMC was defrosted. 1,5–2 x 10^6^ of PBMC were stained with a cocktail of CD45-V500, CD45RA-BB515, CD3-Alexa Fluor700, CD4-PE-Cy7, CD8-BV786, CD197-BV421 (BD Biosciences), CD28-BV711, CD27-APC and CD57-PE (BioLegend) for 15 min at room temperature, washed twice with PBS, pH 7.8 and subsequently stained with viability dye Zombie NIR (BioLegend). 500.000 events in a live/CD45 stopping gate were collected on an Aurora-5 laser instrument Quadrant gating on CD45RA and CD197 (CCR7) was used to define subsets of the CD4^+^ and CD8^+^ populations: naïve like (CD45RA + CD197+), central memory ((T_CM_, CD45RA^−^CD197^+^), effector memory (T_EM_, CD45RA^−^CD197^-^) and effector memory RA (T_EMRA_, CD45RA + CD197-). The expression of the senescence marker CD57 was assessed within each indicated T cell subset. (Gating strategy is given in Berentschot et al., 2023). Subset determinations from staining C are presented as frequency of CD3^+^ T cells, apart from the CD3^+^ T cells themselves which were expressed as a frequency of the lymphocytes. The monoclonal antibodies used in staining B and C are given in Supplementary Table 1.

### 2.5 Serum analysis

For serum hsCRP, IL-6, IL-7 and sCD25 determinations commercially available ELISAs (Advanced Practical Diagnostics BV, Raadsherenstraat 3, 2300 Turnhout, Belgium) were used (by the manufacturer and according to the manufacturer's protocols).

### 2.6 Statistical analysis

We evaluated the differences in the proportion of T cells and differences in depression scores before and after treatment. These were also compared to the outcomes in the contrast group of 42 patients with MDD who received treatment as usual (TAU) and to the control group of 20 healthy controls (HC). In the tables, the variables are presented as individual values for the depressed CVID patients and as means with standard deviations for the group of CVID patients, MDD-TAU and HC group. In the graphs, T-cell numbers and HAM-D scores are presented as individual values for the depressed CVID patients in a before-after plot, combined graphically in box plots together with the MDD-TAU and HC group.

More detailed individual values regarding T-cell numbers and serological analyses of the CVID patients are stated in the complementary tables. The results of the questionnaires used are presented as scores per questionnaire. To evaluate the proportion of depressed CVID patients and MDD-TAU patients with improvements in clinical scores and in T cell senescence parameters after treatment, exact binomial tests were performed comparing outcomes at baseline versus those after treatment (T = 8 weeks). To assess differences in improvement between the CVID patients receiving Tα1 and the MDD-TAU group, the Barnard test is performed. To evaluate the improvement, clinical thresholds were formulated for both the primary and secondary research outcomes. Regarding T-cell numbers, a clinically relevant improvement is hypothesized as a minimum increase in naive T-cell numbers of at least 10%. Regarding HAM-D scores, a clinically significant improvement was defined as a minimum of 3–5 points decrease in the HDRS17 total score or by the remission of the depressive episode (i.e., a HDRS17 total score ≤7) (Rush et al., 2021; Hengartner and Plöderl, 2022). Statistical significance was stated as a p-value <0.0125 due to Bonferroni correction for multiple testing of four hypotheses. Data analysis and visualization was performed in R version 4.3.0. Figures were made in Graphpad Prism version 9.5.0.

## 3 Results

### 3.1 Depression and immune characteristics of the participants at baseline

*Patients with CVID*. Five participants with a CVID and comorbid depression were included in the Erasmus Medical Center, Immunology outpatient clinic to receive Tα1. The median HDRS-17 score for the Depressed CVID group was 19 (standard deviation, 5) (Table 1). Patient numbers 1 and 2 had the most severe depression (i.e. HDRS-17 score of 24 and 25 respectively) and were already at inclusion on anti-depressive drug treatment in a stable dose regimen. This treatment was marginally effective since patient numbers 1 and 2 still had high HDRS scores despite this treatment. We consider these patients as treatment resistant, since both had already received two cycles of anti-depressant treatment in the past without obvious success. In these two patients the antidepressant drugs were continued during the study, and Tα1 was given as an add-on therapy. The other three patients had relatively mild signs of depression with HDRS-17 scores of 14–16. These patients did not receive anti-depressant drugs.

With regard to psychotherapy, only patient 1 received psychotherapy during the trial (aimed at treating somatization), patient 2 and 3 had received psychotherapy in the past 2 year for previous depressive episodes, in one related to the postpartum period.

*Patients with MDD receiving TAU*. The median HDRS-17 score for the MDD-TAU group was 20 (sd = 4) and 2 (sd = 2) for the HC group (Table 1). All patients received cognitive behavioral psychotherapy, 40 % did not receive any antidepressant pharmacological therapy, 30% received a serotonergic drug only, and 30 % received more than one drug (mostly quetiapine, mirtazapine, venlafaxine, bupropion), almost all in combination with an anti-serotonergic drug.

*Baseline levels of routine immune parameters of the depressed CVID patients*. With regard to the routine determinations of lymphocyte subsets per quantity of blood (Staining A) the majority of the patients had reduced levels of B cells (Table 1, exception patient 3), while patients 1 and 2 had increased CD3^+^CD8^+^ cytotoxic T cells and patient numbers 3 and 4 reduced NK cells (Table 1). These routine determinations were not performed in the here studied MDD and HC groups, but studies of our investigation team on a large group of patients with MDD (Simon et al., 2023; Grosse et al., 2016) have also shown increases in CD8^+^ T cytotoxic cells and reduced NK cells.

*Baseline levels of the differentiation of T helper and T cytotoxic subsets of the depressed CVID patients*. In the FACS assay determining naïve and (senescent) memory CD4^+^ and CD8^+^ cells relative to the number of lymphocytes (Staining C, Supplementary Table 2) it became evident that particularly patient numbers 1 and 2 had signs of premature T cell senescence, i.e. low CD4^+^ and CD8^+^ naïve/memory T cell ratios (due to relatively low naïve and relatively high memory cells) and high percentages of CD4^+^CD57^+^ and CD8^+^CD57^+^ memory populations, i.e. non-responsive senescent memory cells (Table 1, Supplementary Table 2). These observations are in accord with a previous study of ours (Manusama et al., 2023) showing similar senescence characteristics in a larger sample of CVID patients. It must also be noted that in the TAU group 28% of the patients show similar premature senescence characteristics of CD4^+^ and CD8^+^ T cells (Table 1, to be published).

*Baseline levels of functional T helper cell subsets of the depressed CVID patients.* With regard to the functional subtypes of CD4^+^ T helper cells (Staining B, Th1, Th2, Th17 and T reg cells) there was a considerable per patient heterogeneity regarding the levels of these CD4^+^ subpopulations as compared to the healthy control levels (Table 1 and Supplementary Table 3). Nevertheless the five CVID patients showed on average increased levels of Th2 cells (Supplementary Table 3). It is tempting to speculate that the higher Th2 activity is linked to an attempt to increase the activity of the (reduced) B cells to produce antibodies in this antibody deficient population.

The MDD patients of the here studied TAU group did not show significant alterations in these subpopulations (to be published); this is in accord with our experience (Schiweck et al., 2020) with large groups of MDD patients who do not show alterations in these functional subpopulations (provided in subgroups characterized by suicidal ideation and/or severe melancholic depression, 26).

*Baseline levels of peripheral cytokines of the depressed CVID patients*. Regarding the level of the peripheral cytokines/inflammatory compounds, only IL-6 levels were clearly raised in patients 1 and 2 (Supplementary Table 4). In the contrast TAU MDD-group IL-6 levels were not significantly raised at entrance of the study.

*Correlation of depression severity in CVID patients and base line immune abnormalities*. Although numbers are too small for reliable correlation studies, it is noteworthy that the 2 most severely depressed drug-resistant CVID patients had the largest immune abnormalities, i.e. a CD4^+^ and CD8^+^ senescent immune profile and the highest IL-6 levels (Table 1).

### 3.2 Effects of thymalfasin (Tα1) treatment for eight weeks

**Fig. 2:**
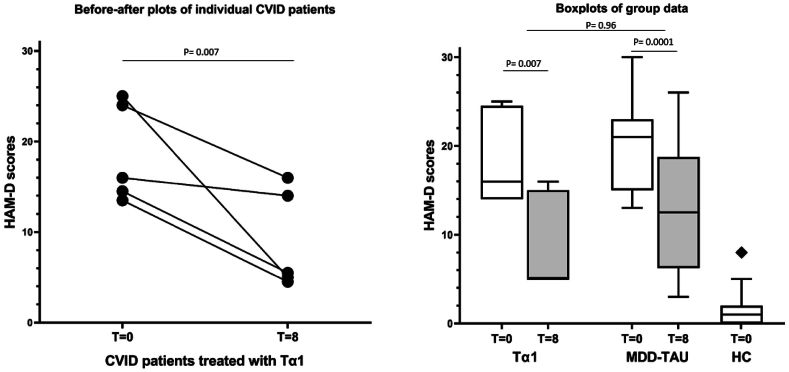
The baseline (T = 0) and 8 week (after treatment) HAMD-17 scores in the 5 depressed CVID patients treated with Thymalfasin (Tα1) given as before-after plot (left panel) and box plots (right panel) with standard deviations (white = baseline, grey = 8 week values). The box plot data for the major depressed (MDD) contrast group treated as usual (TAU) and for the healthy controls (HC) are also given. Significances are as indicated.

*Clinical improvement*. At week eight, all five patients showed a decrease in their depression severity (as assessed by the decrease in the HDRS17 total score) (Fig. 2). Four out of the five patients (80%) experienced a clinically significant improvement of at least five points. When hypothesizing that 20% of patients with MDD in the general population would have this improvement spontaneously, 80% of CVID patients improving with Tα1 is a statistically significant improvement (*p* = 0.007; 95% CI: 0.28 to 0.99) (See also Supplementary Fig. 1). Particularly evident was the improvement on the HDRS17 anxiety subscale score. Of note is that the MDD-TAU group experienced a similar clinical depression score improvement after the treatment as usual (Fig. 2; p < 0.0001).

Fatigue was scored with the Fatigue Severity Scale (FSS). Although all patients had an increased score at baseline (Table 1), none improved on this scale. Also, with regard to the quality-of-life scale (QOLS), patients did not show an overall improvement, although they did on the subitems regarding emotional wellbeing (data not shown).

Tα1 was well tolerated, and serious adverse side or adverse effects of Tα1 treatment were not observed. Mild adverse effects were a transient redness at the injection site. Patient 3 experienced a transient, mild episode of dyspnea three weeks after the start of therapy, which did not require treatment. Supplementary Table 5 gives an overview of reported adverse events of Tα1 treatment, showing that a transient redness is a well-documented mild reaction towards the Tα1 injection. Dyspnea has not been reported.

**Fig. 3:**
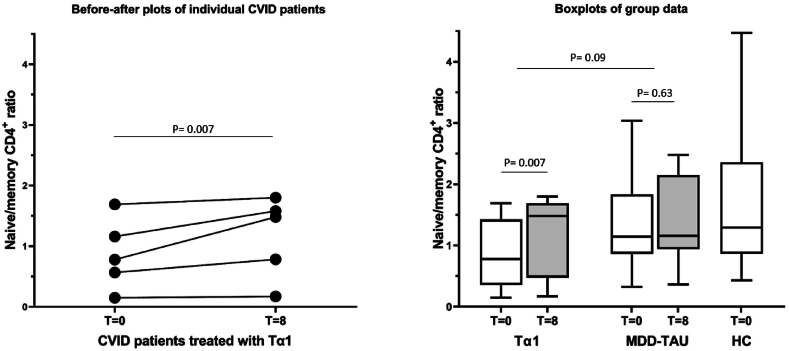
The baseline (T = 0) and 8 week (after treatment) naïve/memory CD4^+^ ratio's in the 5 depressed CVID patients treated with Thymalfasin (Tα1) given as before-after plot (left panel) and box plots (right panel) with standard deviations (white = baseline, grey = 8 week values). The box plot data are also given for the major depressed (MDD) contrast group treated as usual (TAU) and for the healthy controls (HC) (right panel). Significances are as indicated.

**Fig. 4:**
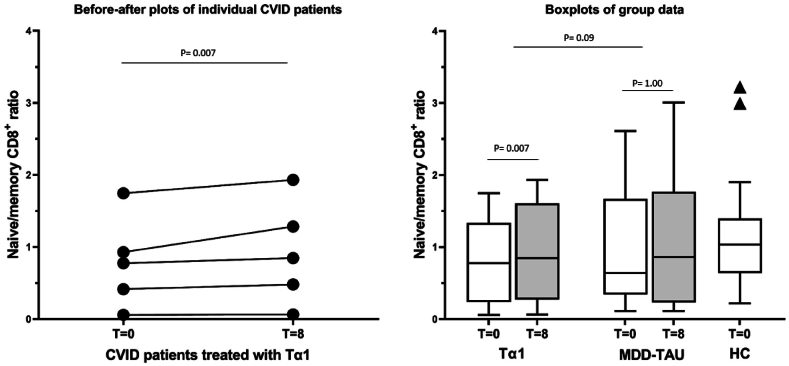
The baseline (T = 0) and 8 week (after treatment) naïve/memory CD8^+^ ratio's in the 5 depressed CVID patients treated with Thymalfasin (Tα1) given as before-after plot (left panel) and box plots (right panel) with standard deviations (white = baseline, grey = 8 week values). The box plot data are also given for the major depressed (MDD) contrast group treated as usual (TAU) and for the healthy controls (HC) (right panel). Significances are as indicated.

*Changes in the senescence of the T helper and T cytotoxic cells*. Treatment with Tα1 decreased the percentage of CD4^+^ T helper memory cells and increased the percentage of CD4^+^ T memory cells in all 5 patients (Supplementary Table 2), which resulted in an increase of the naïve/memory CD4^+^ T helper ratio (Fig. 3). With regard to CD8^+^ cytotoxic T cells, Tα1 treatment increased naïve cells in 4/5 patients, while memory cells decreased in 4/5 patients (not the same patients, Supplementary Table 2). This nevertheless resulted in an increased CD8^+^ T cytotoxic naïve/memory ratio in all 5 patients (Fig. 4). When we apply a binomial analysis on the data before and after treatment statistical significance was reached regarding improvement in CD4^+^ and CD8^+^ naive/memory ratios, when assuming that without therapy one patient would have shown such improvements regarding these T cell senescence parameters spontaneously (see legends figures).

Noteworthy is that, in the contrast MDD-TAU group, there was no indication that treatment as usual did have any such rejuvenating effects on the senescent CD4^+^ and CD8^+^ T helper populations (Fig. 3, Fig. 4).

*Changes in functional T helper cell subsets.* With regard to the Th1, Th2, Th17 and T reg cell populations consistent changes were not induced by thymosin treatment (Supplementary Table 3). Also in the contrast TAU MDD group changes in the Th1, Th2, Th17 and T reg cell populations were not induced by TAU (data not shown).

*Changes in serum cytokine levels.* On average there were no clinically significant increases/decreases in the levels of IL-6, hsCRP, IL-7 and sCD25 in the five patients (Supplementary Table 4). Also significant changes in the average levels of these cytokines were not recorded after treatment in the TAU MDD group (to be published).

Yet, interestingly regarding IL-6 levels, patients 1 and 2 with the highest levels and the highest depression scores, showed clear reductions after eight weeks treatment; patient 1 showed a reduction of IL-6 from 5.9 to 4.7 pg/L and patient 2 from 11.0 to 7.3 pg/L (Supplementary Table 4). Also, the other two patients with measurable IL-6 levels (patient 4 and 5) showed reductions (Supplementary Table 4).

### 3.3 Wash out period of eight weeks

Patient numbers 1 and 2 experienced a serious setback with aggravation of their depression in the wash out period of eight weeks in which the patients did not receive T cell stimulating therapy anymore, while the three other patients with milder depression continued to improve during this period (Complimentary Fig. 1).

Also, all of the above described improvements in the naïve/memory CD4^+^ and CD8^+^ T cell populations for the 5 CVID patients did not continue in 2/5 patients, i.e. patient 3 and 4 (See T3 values in the supplementary Table 2).

## 4 Discussion

This study reports on a non-placebo controlled small trial on the effects of thymus hormone (Thymalfasin, Tα1) carried out in five cases of CVID with a mild to severe depressive episode (HDRS score >12). Two patients with the most severe depression were on antidepressive drugs (but still had considerable HDRS scores of 24 and 25), the other 3 milder cases (scores of 14–16) were not currently treated for their depression.

All five depressed CVID patients showed an improvement of the HAMD score after eight weeks of thymus hormone treatment (median decrease 65%), one of the two most severe cases showing the strongest improvement (decreases 80%). Clinical improvements in the depressed CVID group were similar to those found in the contrast group of MDD patients treated as usual.

Immunologically, all treated patients showed an increase of the naïve/memory CD4^+^ and CD8^+^ T cell ratio. We explain this increase as caused by a stimulation of the production of naïve CD4^+^ and CD8^+^ T cells in the thymus (or thymus related tissues) by Tα1, a well-known effect of the hormone (King and Tuthill, 2016). The T cell state of the two most severe and strongly responding depressed CVID patients improved in particular in this respect.

Interestingly, all CVID patients with measurable blood IL-6 levels (4 of the 5 patients), showed decreases after Tα1 treatment. The two most severely depressed CIVD patients had the highest IL-6 levels and did respond the strongest with a reduction in serum IL-6. Serum IL-6 is known to be upregulated in patients with a depression, and it is considered a sign of so-called low grade inflammation (Köhler et al., 2018; Arteaga-Henríquez et al., 2019). IL-6 is capable of entering the brain via a special entry route (Capuron and Miller, 2011) and capable of inducing inflammatory abnormalities affecting growth and mood regulating function of the cortico-limbic system (Chen et al., 2024).

Patients with MDD treated with an antidepressant regimen as usual and used as a contrast group, although showing clinical improvement, did not show increases in the naïve/memory CD4^+^ and CD8^+^ T cell ratios. This, and also the observation that the two most severely depressed CVID cases thymus hormone had the lowest naïve/memory CD4^+^ and CD8^+^ T cell ratios, and that these ratio's improved together with the depression during Thymalfasin treatment, suggest to us that the here described thymus hormone induced T cell changes underlie the beneficial effects on mood regulation. However, we cannot exclude that we observed an interaction between Tα1 treatment and confounders (such as e.g. anti-depressant or antibiotic treatment) resulting in the clinical improvement. The sample sizes of the subgroups with these confounders are far too small allowing an analysis of any interaction.

Parallels can be drawn between the outcomes of this study and two recently published studies (Poletti et al., 2024; Leboyer et al., 2024) in which low dose IL-2 (ld IL-2) was used in the treatment of unipolar and bipolar depressed patients. The ld IL-2 treatment expanded the percentage of naive CD4^+^ and CD8^+^ T cells, T regulatory and T helper 2 cells and significantly potentiated the antidepressant response to treatment as usual in both study groups. Thus, also in the ld IL-2 treatment study increases in the naïve CD4^+^ and CD8^+^ T cells coincided with an anti-depressive effect.

Regarding the mechanism via which thymus hormone and other T cell stimulatory agents, such as ld IL-2, might improve the depression of patients, we like to believe that the newly created naïve CD4^+^ and CD8^+^ T cells harbor immune cells playing a role in the regulation of the structure and the function of components of the limbic system involved in mood regulation. In preclinical mouse studies, it has been shown that T cells and in particular Th17 and T regulator cells are important in mood regulation by the limbic system (see introduction, and Poletti et al. (2017), Beurel et al. (2018)). However, in the here reported study, thymus hormone treatment did not influence the level of Th17 and T regulator cells, but perhaps other induced and yet undefined “mood-regulating” T cell populations might have played a role.

It is also possible that the newly created naïve CD4^+^ and CD8+T cells are not involved in limbic system support, but strengthen the defense against microbes which might be involved in inducing depression. Chronic CMV (Ford and Savitz, 2023) and toxoplasma infection (Savitz and Yolken, 2023) have been postulated to play a role in depression development.

Furthermore thymus hormone might have acted via dampening IL-6 mediated inflammatory mechanisms in the brain. Thymus hormone reduced the serum IL-6 level in the 4 patients with detectable levels. Correction of the low grade inflammatory state via anti-inflammatory agents is a well-known and effective intervention for MDD; a recent meta-analysis showed several inflammatory agents to be effective (Köhler-Forsberg et al., 2019). In mouse models of LPS-induced chronic pain and impaired hippocampal neurogenesis Tα1 treatment blocked the effects of inflammation (Wang et al., 2017).

Also the gut microbiome has recently gained attention in the pathogenesis of mood disorders (Donoso et al., 2023), and evidence is accumulating that an aging microbiome (Hasavci and Blank, 2022) and microbiome induced alterations in T cell state and function (Manchia et al., 2020) play a role in depression. Thymus hormones might modulate these interactions.

Last but not least, non-immune and direct effects of thymus hormone on the brain might have played a role as well in the here described anti-depressant effects. A precursor of thymosin alpha-1 (prothymosin alpha) is expressed in the neuronal nuclei in the brain, and is involved in multiple functions, such as chromatin remodeling, transcriptional regulation, cell proliferation, and survival (Ueda et al., 2017). It can thus be envisaged that treatment with Tα-1 influences the levels of this pro-hormone.

After the eight weeks thymus hormone therapy patients were left untreated for eight weeks. In this wash out period the depression severity returned more or less to the original situation in the severely depressed patients 1 and 2, while in the milder depressed patients 3, 4 and 5 the improvement continued. Regarding the immune effects, the increase in naïve/memory CD4+and CD8^+^ T cell ratios were by and large not sustained, while also IL-6 levels returned to pre-treatment values during the wash out. The data thus indicate that longer periods of treatment might be necessary.

It is clear from this preliminary report with only 5 patients successfully treated with Thymalfasin, that confirmation studies are required. One of the major limitations of this study is the non-placebo-controlled design of the study, limiting a detailed statistical evaluation. Therefore, we only presented the results of comparison in proportions as the sample size was very small. Due to this small sample size, the beneficial effect of Tα-1 treatment could not be statistically powered in relation to TAU. Also, as for depression, well defined clinical thresholds for treatment success are described, this is not the case regarding the improvement of T-cell subset counts. Therefore, our data can only be considered as preliminary.

## 5 Conclusions

Taken together, this study suggests a possible beneficial effect of thymus hormone treatment in the management of depression in CVID patients by direct or indirect manner. The hormone treatment (in)directly diminished the HDRS scores (in a similar fashion as TAU in a group of patients with MDD). Immunologically, thymus hormone increased naïve/memory T cell ratio's and decreased signs of low grade inflammation. These data therefore urge for further thymus hormone therapy studies in larger placebo-controlled trials on patients with a major depression, the more since adverse reactions to thymus hormone are negligible (see Supplementary Table 5). Also because preclinical studies in a mouse model of depression showed beneficial effects of treatment with Tα1, improving chronic stress induced anhedonia, anxiety and poor exploratory behaviors. In the model the low levels of CD4^+^ T cells induced by chronic stress (amongst others) improved upon Tα1 treatment too (Cai, 2023).

## CRediT authorship contribution statement

**Daniël G. Aynekulu Mersha:** Writing – original draft, Visualization, Investigation, Formal analysis, Data curation. **Sarah E. Fromme:** Writing – review & editing, Supervision, Conceptualization. **Frank van Boven:** Writing – review & editing, Methodology, Formal analysis, Data curation. **Gara Arteaga-Henríquez:** Writing – review & editing, Supervision, Conceptualization. **Annemarie Wijkhuijs:** Writing – review & editing, Validation, Investigation, Data curation. **Marianne van der Ent:** Writing – review & editing, Supervision. **Raf Bergmans:** Writing – review & editing, Conceptualization. **Bernard T. Baune:** Writing – review & editing, Conceptualization. **Hemmo A. Drexhage:** Writing – review & editing, Writing – original draft, Supervision, Methodology, Conceptualization. **Virgil Dalm:** Writing – review & editing, Supervision, Investigation, Conceptualization.

## Conflicts of interest

HAD is the coordinator of the EU-MOODSTRATIFICATION project and declares no further potential conflict of interest. All other authors declare no potential conflicts of interest.

## Declaration of competing interest

The authors declare the following financial interests/personal relationships which may be considered as potential competing interests: Hemmo A. Drexhage reports financial support was provided by Horizon Europe. If there are other authors, they declare that they have no known competing financial interests or personal relationships that could have appeared to influence the work reported in this paper.

## Data Availability

Data will be made available on request.
