# Supplementary material for: Indications for an antidepressive effect of thymosin alpha-1 in a small open-label proof of concept study in common variable immune deficiency patients with depression
Source: Brain Behav Immun Health. 2025 Jan 2;43:100934. doi: 10.1016/j.bbih.2024.100934 (PMC11762651; doi:10.1016/j.bbih.2024.100934)
Supplement: Multimedia component 1 [file mmc1.docx]

**Supplementary Table 1. Details on the monoclonal antibodies used this study.**

| **Antigen** | **Fluorochrome** | **Clone** | **Brand** | **Cat. No.** | **Working dilution** | **μl/test** |
| --- | --- | --- | --- | --- | --- | --- |
| **Staining B** | | | | | | |
| CD45RO | FITC | UCHL1 | BD Biosciences | 555492 | undiluted | 20.0 |
| CD3 | APC-H7 | SK7 | BD Biosciences | 560176 | undiluted | 5.0 |
| CD4 | PerCP-Cy5.5 | SK3 | BD Biosciences | 566923 | undiluted | 5.0 |
| CD25 | APC | 2A3 | BD Biosciences | 340907 | undiluted | 2.5 |
| IL-4 | PE-Cy7 | 8D4-8 | Thermo Fisher Scientific | 25-7049-82 | undiluted | 1.3 |
| IFN-y | Horizon V500 | B27 | BD Biosciences | 561980 | undiluted | 2.5 |
| IL-17A | BV421 | BL168 | BioLegend | 512322 | undiluted | 3.3 |
| FoxP3 | PE | 259D/C7 | BD Biosciences | 560046 | undiluted | 15.0 |
| **Staining C** | | | | | | |
| CD45 | V500 | HI30 | BD Biosciences | 560777 | undiluted | 2.5 |
| CD45RA | BB515 | HI100 | BD Biosciences | 564552 | undiluted | 0.5 |
| CD3 | Alexa Fluor700 | UCHT1 | BD Biosciences | 557943 | undiluted | 2.5 |
| CD4 | BUV805 | SK3 | BD Biosciences | 612887 | undiluted | 2.5 |
| CD8 | BUV395 | RPA-T8 | BD Biosciences | 563795 | undiluted | 2.5 |
| CD197 | BV421 | 2-L1-A | BD Biosciences | 566743 | undiluted | 5 |
| CD28 | BV711 | 28.2 | BioLegend | 302948 | undiluted | 5 |
| CD27 | APC | M-T271 | BioLegend | 356410 | undiluted | 1 |
| CD57 | PE | HNK-1 | BioLegend | 359612 | undiluted | 2.5 |

**Supplementary Table 2.**

Frequencies of CD3+ T cells (% of CD45+ cells) and T cell subsets (% of CD3+ T cells) of the 5 individual depressed CVID patients at visit T1, T2 and T3 as compared to the average value (plus standard deviation of the healthy controls (HC, n=14). Bold and underlined are T1 values considerably decreased or increased as compared to the healthy controls (average plus or minus ⁓2X the standard deviation; outcomes were not tested statistically).

**Supplementary Table 3.**

Frequencies of Th1, Th2, Th17 and T reg cell subsets (as a % of lymphocytes) of the 5 individual depressed CVID patients at visit T0, T8 and T16 as compared to the average value (plus standard deviation of the healthy controls (HC, n=14). Bold and underlined are individual values decreased or increased as compared to the healthy control values (average plus or minus ⁓1X the standard deviation, n=16). Also the average and standard deviation of the 5 depressed CVID patients is shown; as compared to the healthy control values the proportions of Th2 and Th1 cells are increased in the lymphocyte fractions of the patients (the Th2 cells statistically significantly). There are no clear patterns of increases or decreases of the populations in relation to Thymalfasin treatment.

**Supplementary table 4.**

Serum levels of hsCRP, IL-6, IL7 and sCD25 of the 5 individual CVID patients with depression at the T1, T2 and T3 visit as compared to the average values (and standard deviations) of healthy controls (HC, n=18). Bold and underlined are the T1 IL-6 levels which were largely increased as compared to the healthy control values (average plus 2X the standard deviation). Reductions in IL-6 Levels were seen at T2 in all patients with measurable levels.

**Supplementary Table 5:**

| **Subject Type** | **Number of Subjects1** | **Dose Range (mg)2** | **Duration/ Frequency** | **Route** | **Adverse Experiences Reported as Related to Tα_1_** |
| --- | --- | --- | --- | --- | --- |
| Normal volunteers (single-dose, multiple-dose, bioequivalence) | 98 | 0.8-16 | QD up to 7 days; BIW up to 15 weeks | s.c. | Pain at the injection site, which was mild and resolved after less than 30 minutes |
| Acute infections (severe sepsis; fungal, viral, or bacterial infections) | 872 | 1.6-3.2 | BID up to 12 days; QD for up to 16 weeks | s.c. | None reported |
| Hepatic viral disease (CHB; CHC) | 1,969 | 1.0-3.2 | QD up to 2 weeks; TIW up to  6 months; BIW up  to 12 months | s.c. | Subject deaths attributed to underlying disease; abnormal renal function was seen in 3 subjects and abdominal pain, anemia, fever, hernia, and pancreatitis was seen in 2 subjects; most AEs possibly related were in lower frequency in treated groups; rash reported as related in 2 subjects; nervous system SAEs seen in 6 subjects; 2 subjects had TSH abnormalities; 1 subject attempted suicide; 1 subject gave birth to a baby with esophageal atresia; 1AE, nipple pain, was considered related |
| HIV | 58 | 0.4-3.2 | QD up to  10 weeks; BIW up to 1 year | s.c. | None reported |
| Cancer  (mostly melanoma, HCC, NSCLC; also  gastric, breast, pancreatic, stomach, colon, rectal | 1,006 | 1.0-6.4 | QD up to  3 months; BIW up to 1 year | s.c. | Subject deaths attributed to underlying disease; nipple pain reported as related; AEs no greater in treated versus control subjects; possibly related AEs and SAEs |
| Vaccine augmentation (influenza vaccine  in geriatric subjects;  influenza and hepatitis vaccine in hemodialysis subjects) | 416 | 1.6-6.4 | Once per week for 2 weeks; BIW up to 6 weeks | s.c. | Minor local discomfort at the site of injection |
| DiGeorge anomaly, primary immunodeficiency | 11 | 40 ug/ kg – 1.6 | BIW up to 6 months | s.c. | None reported |
| **Total subjects treated with Tα_1_** | **4,430** |  | | | |

1 Total estimated number of subjects treated with Tα_1_ in clinical investigational studies. Tα_1_ is

commercially available in certain countries in Asia, the Middle East and Latin America, but this table includes only persons treated in clinical studies outlined in the investigator’s brochure

2 When appropriate, doses were converted to mg per person, using a range of 1.6-1.7 m2 per person

Abbreviations: AZT = azidothymidine; BIW = twice per week; CHB = chronic hepatitis B; CHC = chronic hepatitis C; HCC = hepatocellular carcinoma; HIV = human immunodeficiency virus; IFN = interferon; i.m. = intramuscular; m = meter; mg = milligram; s.c. = subcutaneous; NSCLC = non-small cell lung cancer; Tα_1_ = thymosin alpha 1; TIW = three times per week; QD = every day; wks = weeks; yr = year

A summary of important safety findings seen with Tα_1_ use in humans is shown. The Table includes all known information from 83 studies (discussed in further detail below) conducted under US corporate INDs, physician-sponsored INDs, local clinical experience trials in all countries, and post-marketing use, including the use of Tα_1_ for any indication regardless of sponsorship, and shows that Tα_1_ has a clinically acceptable safety profile. The majority of adverse experiences have been assessed as mild to moderate in severity, and consisted primarily of injection-site pain (includes burning) and erythema, as well as fever (pyrexia), nausea, and flu-like symptoms.

Very few Adverse Events (AEs) have been considered related to Tα_1_ or occurred in higher frequency in Tα_1_-treated subjects than in control or placebo subjects:

- Pain at the injection site, which was mild and resolved after less than 30 minutes, has been reported in several studies with Tα_1_
- In a study of chronic hepatitis B with decompensated liver disease, abnormal renal function was seen in 3 of the 1969 subjects and abdominal pain, anemia, fever, hernia, and pancreatitis was seen in 2 of the 1969 subjects (US HBV Monotherapy Study in Decompensated Liver Disease)
- Two subjects with chronic hepatitis C reported an SAE of rash which was attributed to Tα_1_ (Italy phase 3 STI 1474-DM-03-004; US phase 3 Tα_1_-CHC-2K0804)
- Two subjects with chronic hepatitis C developed ascites, while only 1 subject on placebo did so US phase 3 Tα_1_-CHC-2K0804)
- Two subjects with chronic hepatitis C receiving Tα_1_ in combination with interferon-alpha 2b had thyroid stimulating hormone abnormalities (US phase 3 CHC study; Sherman 1998); this has been observed with interferon alone in other studies, but it is possible that the risk of this may have been increased in the setting of Tα_1_ combined with interferon
- One AE, nipple pain, was considered possibly or probably related to Tα_1_, as the rate was 15% in the Tα_1_ treatment arm but none occurred in the control arm in a study of subjects with hepatocellular carcinoma being treated with Tα_1_in combination with TACE (US Phase 2 Tα_1_-HCC-2K100)

Regarding Serious Adverse Events(SAEs), they have also very seldomly been reported in studies with Tα_1_:

- One patient with chronic hepatitis B who was using Tα_1_ in association with ursodeoxycholic acid, glycyrrhizin and ribamide, she gave birth to a baby with esophageal atresia (Japan phase 3 CHB study; Iino 2005), a malformation that occurs in 1 of 3000 to 4000 live births
- One subject with chronic hepatitis B using Tα_1_ in a clinical trial attempted suicide **(**US phase 3 Tα_1_-CHC-2K0804); however, this subject had a history of cognitive dysfunction, which might have accounted for this adverse experience
- In subjects with chronic hepatitis B, human immunodeficiency virus and cancer, there have been deaths which were related to the primary disease.

Most of the clinical studies enrolled subjects from 18 to 70 years of age, however Tα_1_ has been evaluated in 11 children ranging in age from <1 year to 16 years, and the vaccine studies included geriatric subjects up to 100 years of age. Table 1 includes audited and unaudited data from all known completed and ongoing studies, including studies by other sponsors dating back to 1982. Subject numbers represent the best approximation available.

cases, to a subject’s death. Thus, treated subjects should be monitored carefully for possible intensification of the disease state.

*Conclusion:* Tα_1_ has demonstrated a clinically very acceptable safety profile with few risks to the target patient population. Tα_1_ has been given to persons who are immune-compromised for various reasons, including the elderly, persons on hemodialysis with end-stage renal disease, children with primary immunodeficiencies, persons with chronic viral infections including HIV, people with cancer being given chemotherapy and radiation, and people suffering from severe sepsis. Even in these compromised subjects, Tα_1_ has shown very few adverse events and has not been associated with required dose-reductions or treatment termination. The accumulated animal and human data, including toxicology data and pharmacokinetic data, do not suggest any serious safety signals.

Supplementary Figure 1. Boxplots of HDRS-17 scores in the five separate CVID-patients at baseline, after 8 weeks treatment and 8 weeks wash-out
